# Simple Strategies in Multi-Objective MDPs

**DOI:** 10.1007/978-3-030-45190-5_19

**Published:** 2020-03-13

**Authors:** Florent Delgrange, Joost-Pieter Katoen, Tim Quatmann, Mickael Randour

**Affiliations:** 8 Johannes Kepler University, Linz, Austria; 9 University of Birmingham, Birmingham, UK; 10 RWTH Aachen University, Aachen, Germany; 11 UMONS – Université de Mons, Mons, Belgium

## Abstract

We consider the verification of multiple expected reward objectives at once on Markov decision processes (MDPs). This enables a trade-off analysis among multiple objectives by obtaining a Pareto front. We focus on strategies that are easy to employ and implement. That is, strategies that are pure (no randomization) and have bounded memory. We show that checking whether a point is achievable by a pure stationary strategy is NP-complete, even for two objectives, and we provide an MILP encoding to solve the corresponding problem. The bounded memory case is treated by a product construction. Experimental results using Storm and Gurobi show the feasibility of our algorithms.

## 1 Introduction

*MDPs.* Markov decision processes (MDPs) [4,3] are a key model in stochastic decision making. The classical setting involves a system subject to a stochastic model of its environment, and the goal is to synthesize a system controller, represented as a *strategy* for the MDP, ensuring a given level of *expected performance*. Tools such as Prism [30] and Storm [16] support MDP model checking.

*Multi-objective MDPs.* MDPs where the goal is to achieve a *combination* of objectives (rather than just one) are popular in e.g., AI [41] and verification [2]. This is driven by applications, where controllers have to fulfill multiple, potentially conflicting objectives, requiring a *trade-off* analysis. This includes multi-dimension MDPs [14,20,40,13] where weight vectors are aggregated at each step and MDPs where the specification mixes different views (e.g., average and worst case performance) of the same weight [11,8]. With multiple objectives, optimal strategies no longer exist in general: instead, *Pareto-optimal* strategies are considered. The Pareto front, i.e., the set of non-dominated achievable value vectors is usually non-trivial. Elaborate techniques are needed to explore it efficiently, e.g., [23,24].

*Simple strategies.* Another stumbling block in multi-objective MDPs is the complexity of strategies: Pareto-optimal strategies typically need both *memory* and *randomization.* A simple conjunction of reachability objectives already requires randomization and exponential memory (in the number of reachability sets) [40].

† currently affiliated with Vrije Universiteit Brussel.

* Research partially supported by F.R.S.-FNRS Grant n° F.4520.18 (*ManySynth*). Mickael Randour is an F.R.S.-FNRS Research Associate.

Some complex objectives even need infinite memory, e.g., [11,8]. In controller synthesis, strategies requiring randomization and/or (much) memory may not be practical. Limited-memory strategies are required on devices with limited resources [7]. Randomization is elegant and powerful from a theoretical view, but has practical limitations, e.g., it limits reproducibility which complicates debugging. Randomized strategies are also often despised for medical applications [33] and product design – all products should have the same design, not a random one. This motivates to consider the analysis of *simple strategies*, i.e., strategies using no randomization and a limited amount of memory (given as a parameter). While most works study the Pareto front among *all* strategies, we establish ways to explore efficiently the Pareto front among *simple* strategies only.

*Problem statement.* We consider pure (i.e., no randomization) and bounded-memory strategies and study two problems: (a) *achievability* queries – is it possible to achieve a given value vector – and (b) *approximation of the Pareto front.* Considering pure, bounded-memory strategies is natural as randomization can be traded for memory [12]: without randomization, optimal strategies may require arbitrarily large memory, (see Ex. 4). We study mixtures of *expected (accumulated) reward objectives*, covering various studied settings like reachability [20,40], shortest path [39,40,28,9] and total reward objectives [23,24].

*Contributions.* We first consider the achievability problem for pure stationary (i.e., memoryless) strategies and show that finding optimal strategies for multi-objective MDPs is NP-complete, even for two objectives. This contrasts the case of general strategies, where the problem is polynomial-time if the number of objectives is fixed [40]. We provide a *mixed integer linear program* (`MILP`) encoding. The crux lies in dealing with end components. The `MILP` is polynomial in the input MDP and the number of objectives. Inspired by [22], we give an alternative `MILP` encoding which is better suited for total reward objectives. To approximate the Pareto front under pure stationary strategies, we solve multiple `MILP` queries. This iteratively divides the solution space into achievable and non-achievable regions. Bounded-memory strategies are treated via a product construction. Our approach works for finite *and* infinite expected rewards.

*Practical evaluation.* We successfully compute Pareto fronts for 13 benchmarks using our implementation in Storm, exploiting the `MILP` solver Gurobi. Despite the hard nature of the problem, our experiments show that Pareto fronts for models with tens of thousands of states can be successfully approximated.

*Related work.* NP completeness for discounted rewards under pure strategies was shown in [14]. [19] claims that this generalizes to PCTL objectives but no proof is given. [42] treats multi-objective bounded MDPs whose transition probabilities are intervals. A set of Pareto optimal policies is computed using policy iteration and an efficient heuristic is exploited to compute a set of mutually non-dominated policies that are likely to be Pareto optimal. Pure stationary Pareto optimal strategies for discounted rewards are obtained in [44] using value-iteration but is restricted to small MDPs where all probabilities are 0 or 1. In [34],Tchebycheff-optimal strategies for discounted rewards are obtained via an LP approach; such strategies minimize the distance to a reference point and are not always pure.

## 2 Preliminaries

For a finite set $\Omega$, let $Dist(\Omega) = \{\mu\colon \Omega \to [0,1] \mid \sum_{\omega\in\Omega} \mu(\omega) = 1\}$ be the set of probability distributions over $\Omega$ with support $supp(\mu) = \{\omega \in \Omega \mid \mu(\omega) > 0\}$. We write $\mathbb{R}_{\geq 0} = \{|x| \mid x \in \mathbb{R}\}$ and $\mathbb{R}_\infty = \mathbb{R} \cup \{\infty\}$ for the non-negative and extended real numbers, respectively. $\mathbf{1}^\ell = \langle 1, \ldots, 1\rangle$ denotes the vector of size $\ell \in \mathbb{N}$ with all entries 1. We just write $\mathbf{1}$ if $\ell$ is clear. Let $\boldsymbol{p}[\![i]\!]$ denote the $i^{th}$ entry and $\boldsymbol{p} \cdot \boldsymbol{p}'$ the dot product of $\boldsymbol{p}, \boldsymbol{p}' \in (\mathbb{R}_\infty)^\ell$. $\boldsymbol{p} \leq \boldsymbol{p}'$, $\boldsymbol{p} + \boldsymbol{p}'$, and $|\boldsymbol{p}|$ are entry-wise. For Boolean expression *cond*, let $[cond] = 1$ if *cond* is true and $[cond] = 0$ otherwise.

### 2.1 Markov Decision Processes, Strategies, and End Components

**Definition 1 (Markov decision process [36]).** *A* Markov decision process *(MDP) is a tuple* $\mathcal{M} = \langle S, Act, \mathbf{P}, s_I\rangle$ *with finite set of states* $S$*, initial state* $s_I \in S$*, finite set of actions* $Act$*, and transition function* $\mathbf{P}\colon S \times Act \times S \to [0,1]$ *with* $\sum_{s'\in S} \mathbf{P}(s, \alpha, s') \in \{0,1\}$ *for all* $s \in S$ *and* $\alpha \in Act$*.*

We fix an MDP $\mathcal{M} = \langle S, Act, \mathbf{P}, s_I\rangle$. Intuitively, $\mathbf{P}(s, \alpha, s')$ is the probability to take a transition from $s$ to $s'$ when choosing action $\alpha$. An infinite path in $\mathcal{M}$ is a sequence $\pi = s_0\alpha_1 s_1\alpha_2 \cdots \in (S\times Act)^\omega$ with $\mathbf{P}(s_i, \alpha_{i+1}, s_{i+1}) > 0$ for all $i \in \mathbb{N}$. We write $\pi[i] = s_i$ for the $(i{+}1)$th state visited by $\pi$ and define the length of $\pi$ as $|\pi| = \infty$. A finite path is a finite prefix $\hat{\pi} = s_0\alpha_1 \ldots \alpha_n s_n$ of infinite path $\pi$, where $last(\hat{\pi}) = s_n \in S$, $|\hat{\pi}| = n$ and $\hat{\pi}[i] = s_i$ for $i \leq n$. The set of finite (infinite) paths in $\mathcal{M}$ is denoted by $Paths^{\mathcal{M}}_{\mathrm{fin}}$ ($Paths^{\mathcal{M}}_{\mathrm{inf}}$). The *enabled actions* at a state $s \in S$ are given by the set $Act(s) = \{\alpha \in Act \mid \exists\, s' \in S\colon\ \mathbf{P}(s, \alpha, s') > 0\}$. We assume $Act(s) \neq \emptyset$ for all $s$. If $|Act(s)| = 1$ for all $s \in S$, $\mathcal{M}$ is called a *Markov Chain (MC)*. We write $\mathcal{M}_s$ for the MDP obtained by replacing the initial state of $\mathcal{M}$ by $s \in S$. For $s \in S$ and $\alpha \in Act$, we define the set of successor states $succ(s, \alpha) = \{s' \mid \mathbf{P}(s, \alpha, s') > 0\}$. For $s' \in S$, the set of predecessor state-action pairs is given by $pre(s') = \{\langle s, \alpha\rangle \mid \mathbf{P}(s, \alpha, s') > 0\}$. For a set $\mathcal{E} \subseteq S \times Act$, we define $S[\![\mathcal{E}]\!] = \{s \in S \mid \exists\, \alpha\colon\ \langle s, \alpha\rangle \in \mathcal{E}\}$, $Act[\![\mathcal{E}]\!] = \{\alpha \in Act \mid \exists\, s\colon\ \langle s, \alpha\rangle \in \mathcal{E}\}$, and $\mathbf{P}[\![\mathcal{E}]\!](s, \alpha, s') = [\langle s, \alpha\rangle \in \mathcal{E}] \cdot [s' \in S[\![\mathcal{E}]\!]] \cdot \mathbf{P}(s, \alpha, s')$. We say $\mathcal{E}$ is *closed* for $\mathcal{M}$ if $\forall\ \langle s, \alpha\rangle \in \mathcal{E}\colon\ \alpha \in Act(s)$ and $succ(s, \alpha) \subseteq S[\![\mathcal{E}]\!]$.

**Definition 2 (Sub-MDP).** *The* sub-MDP *of* $\mathcal{M}$*, closed* $\mathcal{E} \subseteq S \times Act$*, and* $s \in S[\![\mathcal{E}]\!]$ *is given by* $\mathcal{M}[\![\mathcal{E}, s]\!] = \langle S[\![\mathcal{E}]\!], Act[\![\mathcal{E}]\!], \mathbf{P}[\![\mathcal{E}]\!], s\rangle$*. We also write* $\mathcal{M}[\![\mathcal{E}]\!]$ *for the sub-MDP* $\mathcal{M}[\![\mathcal{E}, s]\!]$ *and an arbitrary state* $s \in S[\![\mathcal{E}]\!]$*.*

**Definition 3 (End Component).** *A non-empty set* $\mathcal{E} \subseteq S \times Act$ *is an* end component (EC) *of* $\mathcal{M}$ *if* $\mathcal{E}$ *is closed for* $\mathcal{M}$ *and for each pair of states* $s, s' \in S[\![\mathcal{E}]\!]$ *there is a finite path* $\hat{\pi} \in Paths^{\mathcal{M}[\![\mathcal{E}]\!]}_{\mathrm{fin}}$ *with* $\hat{\pi}[0] = s$ *and* $last(\hat{\pi}) = s'$*. An EC* $\mathcal{E}$ *is* maximal*, if there is no other EC* $\mathcal{E}'$ *with* $\mathcal{E} \subsetneq \mathcal{E}'$*. The set of all maximal end components of* $\mathcal{M}$ *is* $MECS(\mathcal{M})$*.*

The maximal ECs of a Markov chain are also called *bottom strongly connected components (BSCCs)*. A strategy resolves nondeterminism in MDPs:

**Definition 4 (Strategy).** *A (general)* strategy *for MDP* $\mathcal{M}$ *is a function* $\sigma\colon \mathit{Paths}^{\mathcal{M}}_{\mathrm{fin}} \to \mathit{Dist}(\mathit{Act})$ *with* $\mathit{supp}(\sigma(\hat{\pi})) \subseteq \mathit{Act}(\mathit{last}(\hat{\pi}))$ *for all* $\hat{\pi} \in \mathit{Paths}^{\mathcal{M}}_{\mathrm{fin}}$.

Let $\sigma$ be a strategy for $\mathcal{M}$. Intuitively, $\sigma(\hat{\pi})(\alpha)$ is the probability to perform action $\alpha$ after observing history $\hat{\pi} \in \mathit{Paths}^{\mathcal{M}}_{\mathrm{fin}}$. A strategy is *pure* if all histories are mapped to *Dirac distributions*, i.e., the support is a singleton. A strategy is *stationary* if its decisions only depend on the current state, i.e., $\forall\, \hat{\pi}, \hat{\pi}' \in \mathit{Paths}^{\mathcal{M}}_{\mathrm{fin}}\colon \mathit{last}(\hat{\pi}) = \mathit{last}(\hat{\pi}')$ implies $\sigma(\hat{\pi}) = \sigma(\hat{\pi}')$. We often assume $\sigma\colon S \to \mathit{Dist}(\mathit{Act})$ for stationary and $\sigma\colon S \to \mathit{Act}$ for pure stationary strategies $\sigma$. Let $\Sigma^{\mathcal{M}}$ and $\Sigma^{\mathcal{M}}_{\mathrm{PS}}$ be the sets of general and pure stationary strategies, respectively. A set of paths $\Pi \subseteq \mathit{Paths}^{\mathcal{M}}_{\mathrm{inf}}$ is *compliant* with $\sigma \in \Sigma^{\mathcal{M}}$ if for all $\pi = s_0\alpha_1 s_1 \cdots \in \Pi$ and prefixes $\hat{\pi}$ of $\pi$ satisfy $\sigma(\hat{\pi})(\alpha_{|\hat{\pi}|+1}) > 0$. The *induced Markov chain* of $\mathcal{M}$ and $\sigma \in \Sigma^{\mathcal{M}}_{\mathrm{PS}}$ is given by $\mathcal{M}^{\sigma} = \mathcal{M}[\![\mathcal{E}^{\sigma}, s_I]\!]$ with $\mathcal{E}^{\sigma} = \{\langle s, \sigma(s)\rangle \mid s \in S\}$.

MDP $\mathcal{M}$ and strategy $\sigma \in \Sigma^{\mathcal{M}}$ induce a probability measure $\mathrm{Pr}^{\mathcal{M}}_{\sigma}$ on subsets $\Pi \subseteq \mathit{Paths}^{\mathcal{M}}_{\mathrm{inf}}$ given by a standard cylinder set construction [4,22]. The expected value of $X\colon \mathit{Paths}^{\mathcal{M}}_{\mathrm{inf}} \to \mathbb{R}_{\infty}$ is $\mathrm{E}^{\mathcal{M}}_{\sigma}(X) = \int_{\pi} X(\pi)\, d\mathrm{Pr}^{\mathcal{M}}_{\sigma}(\{\pi\})$. For $\sigma \in \Sigma^{\mathcal{M}}_{\mathrm{PS}}$, $\mathrm{Pr}^{\mathcal{M}}_{\sigma}$ and $\mathrm{E}^{\mathcal{M}}_{\sigma}$ coincide with the corresponding measures on MC $\mathcal{M}^{\sigma}$.

### 2.2 Objectives

A reward structure $\mathbf{R}\colon S \times \mathit{Act} \times S \to \mathbb{R}_{\geq 0}$ assigns non-negative rewards to transitions. We accumulate rewards on (in)finite paths $\pi = s_0\alpha_1 s_1\alpha_2 \ldots\colon \mathbf{R}(\pi) = \sum_{i=1}^{|\pi|} \mathbf{R}(s_{i-1}, \alpha_i, s_i)$. For a set of goal states $G \subseteq S$, let $\mathbf{R}\Diamond G(\pi) = \mathbf{R}(\hat{\pi})$, where $\hat{\pi}$ is the smallest prefix of $\pi$ with $\mathit{last}(\hat{\pi}) \in G$ (or $\hat{\pi} = \pi$ if no such prefix exists). Intuitively, $\mathbf{R}\Diamond G(\pi)$ is the reward accumulated on $\pi$ until a state in $G$ is reached. A (reward) *objective* has the form $\mathbb{E}_{\sim}(\mathbf{R}\Diamond G)$ for $\sim\, \in \{\geq, \leq\}$. We write $\langle \mathcal{M}, \sigma, p\rangle \models \mathbb{E}_{\sim}(\mathbf{R}\Diamond G)$ iff $\mathrm{E}^{\mathcal{M}}_{\sigma}(\mathbf{R}\Diamond G) \sim p$, i.e., for $\mathcal{M}$ and $\sigma$, the expected accumulated reward until reaching $G$ is at least (or at most) $p \in \mathbb{R}_{\infty}$. We call the objective *maximizing* if $\sim\, = \,\geq$ and *minimizing* otherwise. If $G = \emptyset$ (i.e., $\mathbf{R}\Diamond G(\pi) = \mathbf{R}(\pi)$ for all paths $\pi$), we call the objective a *total reward objective*. Let the reward structure $\mathbf{R}^{G}$ be given by $\mathbf{R}(s, \alpha, s') = [s' \in G]$. Then, $\mathrm{Pr}^{\mathcal{M}}_{\sigma}(\Diamond G) = \mathrm{E}^{\mathcal{M}}_{\sigma}(\mathbf{R}^{G}\Diamond G)$ for every $\sigma \in \Sigma^{\mathcal{M}}$, where $\Diamond G \subseteq \mathit{Paths}^{\mathcal{M}}_{\mathrm{inf}}$ denotes the set of paths that visit a state in $G$. We use $\mathbb{P}_{\sim}(\Diamond G)$ as a shortened for $\mathbb{E}_{\sim}(\mathbf{R}^{G}\Diamond G)$ and call such an objective a *reachability objective*.

**Definition 5 (Multi-objective query).** *For MDP* $\mathcal{M}$*, an* $\ell$-dimensional multi-objective query *is a tuple* $\mathcal{Q} = \langle \psi_1, \ldots, \psi_\ell\rangle$ *of* $\ell$ *objectives* $\psi_j = \mathbb{E}_{\sim_j}(\mathbf{R}_j\Diamond G_j)$.

Each objective $\psi_j$ considers a different reward structure $\mathbf{R}_j$. The MDP $\mathcal{M}$, strategy $\sigma$, and point $\boldsymbol{p} \in (\mathbb{R}_{\infty})^{\ell}$ satisfy a multi-objective query $\mathcal{Q} = \langle \psi_1, \ldots, \psi_\ell\rangle$ (written $\langle \mathcal{M}, \sigma, \boldsymbol{p}\rangle \models \mathcal{Q}$) iff $\forall\, j\colon\ \langle \mathcal{M}, \sigma, \boldsymbol{p}[\![j]\!]\rangle \models \psi_j$. Then, we also say $\sigma$ *achieves* $\boldsymbol{p}$ and call $\boldsymbol{p}$ *achievable*. Let $\mathit{Ach}^{\mathcal{M}}(\mathcal{Q})$ ($\mathit{Ach}^{\mathcal{M}}_{\mathrm{PS}}(\mathcal{Q})$) denote the set of points achieved by a general (pure stationary) strategy. The *closure* of a set $P \subseteq (\mathbb{R}_{\infty})^{\ell}$ with respect to query $\mathcal{Q}$ is $\mathit{cl}^{\mathcal{Q}}(P) = \big\{\boldsymbol{p} \in (\mathbb{R}_{\infty})^{\ell} \mid \exists\, \boldsymbol{p}' \in P\colon\ \forall\, j\colon\ \boldsymbol{p}'[\![j]\!] \sim_j \boldsymbol{p}[\![j]\!]\big\}$. For $\boldsymbol{p}, \boldsymbol{p}' \in (\mathbb{R}_{\infty})^{\ell}$, we say that $\boldsymbol{p}$ *dominates* $\boldsymbol{p}'$ if $\boldsymbol{p}' \in \mathit{cl}^{\mathcal{Q}}(\{\boldsymbol{p}\})$. In this case, $\langle \mathcal{M}, \sigma, \boldsymbol{p}\rangle \models \mathcal{Q}$ implies $\langle \mathcal{M}, \sigma, \boldsymbol{p}'\rangle \models \mathcal{Q}$ for any $\sigma \in \Sigma^{\mathcal{M}}$. We are interested in the Pareto front, which is the set of non-dominated achievable points.

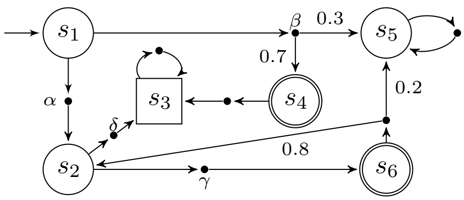

(a) MDP $\mathcal{M}$ with $G_{\circledcirc}=\{s_4, s_6\}$ and $G_{\square}=\{s_3\}$

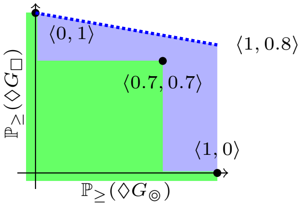

(b) $Ach^{\mathcal{M}}(\mathcal{Q})$ and $Ach^{\mathcal{M}}_{\mathrm{PS}}(\mathcal{Q})$

Figure 1: An MDP and a plot of the pure stationary and general Pareto fronts.

**Definition 6 (Pareto front).** *The* (general) Pareto front *for* $\mathcal{M}$ *and* $\mathcal{Q}$ *is* $Pareto^{\mathcal{M}}(\mathcal{Q}) = \left\{ \boldsymbol{p} \in Ach^{\mathcal{M}}(\mathcal{Q}) \mid \forall \boldsymbol{p}' \in Ach^{\mathcal{M}}(\mathcal{Q})\colon\ \boldsymbol{p} \in cl^{\mathcal{Q}}(\{\boldsymbol{p}'\}) \implies \boldsymbol{p} = \boldsymbol{p}' \right\}$.

The Pareto front is the smallest set $P \subseteq (\mathbb{R}_{\infty})^{\ell}$ with $cl^{\mathcal{Q}}(P) = Ach^{\mathcal{M}}(\mathcal{Q})$. In a similar way, we define the *pure stationary* Pareto front $Pareto^{\mathcal{M}}_{\mathrm{PS}}(\mathcal{Q})$ which only consider points in $Ach^{\mathcal{M}}_{\mathrm{PS}}(\mathcal{Q})$.

*Example 1.* Let $\mathcal{M}$ be the MDP in Fig. 1a and $\mathcal{Q} = \langle \mathbb{P}_{\geq}(\Diamond G_{\circledcirc}), \mathbb{P}_{\geq}(\Diamond G_{\square})\rangle$. A pure stationary strategy choosing $\beta$ at $s_1$ reaches both, $s_4 \in G_{\circledcirc}$ and $s_3 \in G_{\square}$ with probability 0.7 and thus achieves $\langle 0.7, 0.7\rangle$. Similarly, $\langle 0, 1\rangle$ and $\langle 1, 0\rangle$ are achievable by a pure stationary strategy. Point $\langle 1, 0.8\rangle$ is achievable by a non-stationary pure strategy that chooses $\alpha$ at $s_1$, $\gamma$ at the first visit of $s_2$, and $\delta$ in all other cases. Changing this strategy by picking $\gamma$ only with probability 0.5 achieves $\langle 0.5, 0.9\rangle$. Fig. 1b illustrates $Pareto^{\mathcal{M}}_{\mathrm{PS}}(\mathcal{Q})$ (dots), $Ach^{\mathcal{M}}_{\mathrm{PS}}(\mathcal{Q})$ (green area), $Pareto^{\mathcal{M}}(\mathcal{Q})$ (dotted line), and $Ach^{\mathcal{M}}(\mathcal{Q})$ (blue and green area).

## 3 Deciding Achievability

The achievability problem asks whether a given point is achievable.

General Multi-objective Achievability Problem (GMA)

**Input:** MDP $\mathcal{M}$, $\ell$-dimensional multi-objective query $\mathcal{Q}$, point $\boldsymbol{p} \in (\mathbb{R}_{\infty})^{\ell}$
**Output:** Yes iff $\boldsymbol{p} \in Ach^{\mathcal{M}}(\mathcal{Q})$ holds

For GMA, the point can be achieved by a general strategy that can potentially make use of memory and randomization. As discussed earlier, this class of strategies is not suitable for various applications. In this work, we focus on a variant of the achievability problem that only considers pure stationary strategies. Sect. 5 also addresses pure strategies that can store more information from the history, e.g., whether a goal state set has been reached already.

Pure Stationary Multi-objective Achievability Problem (PSMA)

**Input:** MDP $\mathcal{M}$, $\ell$-dimensional multi-objective query $\mathcal{Q}$, point $\boldsymbol{p} \in (\mathbb{R}_{\infty})^{\ell}$
**Output:** Yes iff $\boldsymbol{p} \in Ach^{\mathcal{M}}_{\mathrm{PS}}(\mathcal{Q})$ holds

### 3.1 Complexity Results

GMA is PSPACE hard (already with only reachability objectives) [40] and solvable within exponential runtime [20,23]. To the best of our knowledge, a PSPACE upper bound on the complexity of GMA is unknown. This complexity is rooted in the dimension $\ell$ of the query $\mathcal{Q}$: for fixed $\ell$, the algorithms of [20,23] have polynomial runtime. In contrast, PSMA is NP-complete, even if restricted to 2 objectives.

**Lemma 1.** PSMA *with only reachability objectives is NP-hard.*

*Proof.* The result follows by a reduction from the subset sum problem. Given $n \in \mathbb{N}$, $\boldsymbol{a} \in \mathbb{N}^n$ and $z \in \mathbb{N}$, the subset sum problem is to decide the existence of $\boldsymbol{v} \in \{0,1\}^n$ such that $\boldsymbol{v} \cdot \boldsymbol{a} = z$. This problem is NP-complete [25]. For a given instance of the subset sum problem, we construct the MDP $\mathcal{M}^\star = \langle S, Act, \mathbf{P}, s_I \rangle$ with state space $S = \{s_I, s_1, \dots, s_n, g_1, g_2\}$, actions $Act = \{\alpha, Y, N\}$, and for all $i \in \{1, \dots, n\}$, $\mathbf{P}(s_I, \alpha, s_i) = \frac{\boldsymbol{a}[\![i]\!]}{\mathbf{1} \cdot \boldsymbol{a}}$ and $\mathbf{P}(s_i, Y, g_1) = \mathbf{P}(s_i, N, g_2) = 1$. States $g_1$ and $g_2$ are made absorbing, i.e., $\mathbf{P}(g_1, \alpha, g_1) = \mathbf{P}(g_2, \alpha, g_2) = 1$.

We claim that the PSMA problem for $\mathcal{M}^\star$, $\mathcal{Q} = \langle \mathbb{P}_{\geq}(\lozenge \{g_1\}), \mathbb{P}_{\geq}(\lozenge \{g_2\}) \rangle$, and $\boldsymbol{p} = \left(\frac{z}{\mathbf{1} \cdot \boldsymbol{a}}, 1 - \frac{z}{\mathbf{1} \cdot \boldsymbol{a}}\right)$ answers "yes" iff there is a vector $\boldsymbol{v}$ satisfying the subset sum problem for $n$, $\boldsymbol{a}$ and $z$. Consider the bijection $f \colon \Sigma_{\mathrm{PS}}^{\mathcal{M}^\star} \to \{0,1\}^n$ with $f(\sigma)[\![i]\!] = [\sigma(s_i){=}Y]$ for all $\sigma \in \Sigma_{\mathrm{PS}}^{\mathcal{M}^\star}$ and $i \in \{1, \dots, n\}$. We get $\mathrm{Pr}_\sigma^{\mathcal{M}^\star}(\lozenge \{g_1\}) = \sum_{i=1}^{n} \frac{\boldsymbol{a}[\![i]\!]}{\mathbf{1} \cdot \boldsymbol{a}} [\sigma(s_i){=}Y] = \frac{f(\sigma) \cdot \boldsymbol{a}}{\mathbf{1} \cdot \boldsymbol{a}}$. Moreover, $\mathrm{Pr}_\sigma^{\mathcal{M}^\star}(\lozenge \{g_2\}) = 1 - \mathrm{Pr}_\sigma^{\mathcal{M}^\star}(\lozenge \{g_1\}) = 1 - \frac{f(\sigma) \cdot \boldsymbol{a}}{\mathbf{1} \cdot \boldsymbol{a}}$. It follows that $\sigma$ achieves $\boldsymbol{p}$ iff $f(\sigma)$ is a solution to the instance of the subset sum problem. Our construction is inspired by similar ideas from [14,40].

**Lemma 2 ([14]).** PSMA *with only total reward objectives is NP-hard.*

**Theorem 1.** PSMA *is NP-complete.*

*Proof.* Containment follows by guessing a pure stationary strategy and evaluating it on the individual objectives. This can be done in polynomial time [4]. Hardness follows by either Lemma 1 or 2.

Proofs of Lemmas 1 and 2 only consider 2-dimensional multi-objective queries. Hence, in contrast to GMA, the hardness of PSMA is not due to the size of the query.

**Corollary 1.** PSMA *with only two objectives is NP-complete.*

### 3.2 A Mixed Integer Linear Programming Approach

An MDP $\mathcal{M} = \langle S, Act, \mathbf{P}, s_I \rangle$ has exactly $|\Sigma_{\mathrm{PS}}^{\mathcal{M}}| = \prod_{s \in S} |Act(s)|$ many pure stationary strategies. A simple algorithm for PSMA enumerates all $\sigma \in \Sigma_{\mathrm{PS}}^{\mathcal{M}}$ and checks whether $\langle \mathcal{M}, \sigma, \boldsymbol{p} \rangle \models \mathcal{Q}$ holds. In practice, however, such a brute-force approach is not feasible. For the MDPs that we consider in our experiments in Sect. 6, the number of pure stationary strategies often exceeds $10^{10\,000}$. Instead, our approach is to encode an instance for PSMA as an MILP problem.

| | |
|---|---|
| Mixed Integer Linear Programming Problem (MILP) | |
| **Input:** | $\ell, m, n \in \mathbb{N}$, $\boldsymbol{A} \in \mathbb{Q}^{n \times (\ell+m)}$, $\boldsymbol{b} \in \mathbb{Q}^n$, $\boldsymbol{c} \in \mathbb{Q}^{\ell+m}$ |
| **Output:** | $\begin{cases} \boldsymbol{x} \in \arg\max_{\boldsymbol{x} \in \mathcal{X}} \boldsymbol{c}^T \boldsymbol{x} & \text{if } \mathcal{X} \neq \emptyset \\ \texttt{infeasible} & \text{if } \mathcal{X} = \emptyset \end{cases}$ with $\mathcal{X} = \{\boldsymbol{x} \in \mathbb{Z}^\ell \times \mathbb{R}^m \mid \boldsymbol{A}\boldsymbol{x} \leq \boldsymbol{b}\}$ |

For an MILP instance as above, each of the $n$ rows of the inequation system $\boldsymbol{A}\boldsymbol{x} \leq \boldsymbol{b}$ represent a *constraint* that is linear over the $\ell$ integral and $m$ real-valued *variables* given by $\boldsymbol{x}$. We call the constraints *feasible* if there is a solution to the inequation system. The task is to decide whether the constraints are feasible and if so, find a solution that maximizes a linear optimization function $\boldsymbol{c}^T\boldsymbol{x}$. The optimization function can be omitted if we are only interested in feasibility. MILP is NP-complete [35]. However, tools such as Gurobi [27] and SCIP [26] implement practically efficient algorithms that can solve large instances.

For the rest of this section, let $\mathcal{M} = \langle S, Act, \mathbf{P}, s_I \rangle$, $\mathcal{Q} = \langle \psi_1, \ldots, \psi_\ell \rangle$ with $\psi_j = \mathbb{E}_{\sim_j}(\mathbf{R}_j \Diamond G_j)$, and $\boldsymbol{p} \in (\mathbb{R}_\infty)^\ell$ be an instance for PSMA. We provide a translation of the PSMA instance to an instance for MILP that has a feasible solution iff $\boldsymbol{p} \in Ach^{\mathcal{M}}_{\mathrm{PS}}(\mathcal{Q})$. The MILP encoding considers integer variables to encode a pure stationary strategy $\sigma \in \Sigma^{\mathcal{M}}_{\mathrm{PS}}$. The other variables and constraints encode the expected reward for each objective on the induced MC $\mathcal{M}^\sigma$.

### 3.3 Unichain MDP and Finite Rewards

**Restriction 1 (Unichain MDP).** *MDP $\mathcal{M}$ has exactly one end component.*

**Restriction 2 (Reward Finiteness).** $\mathrm{E}^{\mathcal{M}_s}_\sigma(\mathbf{R}_j \Diamond G_j) < \infty$ *holds for each objective $\psi_j = \mathbb{E}_{\sim_j}(\mathbf{R}_j \Diamond G_j)$, state $s$, and pure stationary strategy $\sigma$.*

For simplicity, we first explain our encoding for unichain MDP with finite reward. Sect. 3.5 lifts Restriction 1 and Sect. 3.6 lifts Restriction 2 with more details given in [17, App. B]. Sect. 3.4 presents an alternative to the encoding of this section, which is smaller but restricted to *total* reward objectives.

Fig. 2 shows the MILP encoding in case Restrictions 1 and 2 hold. We assume $\forall j\colon \boldsymbol{p}[\![j]\!] \neq \infty$ for the point $\boldsymbol{p}$ since (i) $\mathrm{E}^{\mathcal{M}}_\sigma(\mathbf{R}_j \Diamond G_j) \leq \infty$ holds trivially and (ii) $\mathrm{E}^{\mathcal{M}}_\sigma(\mathbf{R}_j \Diamond G_j) \geq \infty$ will never hold due to Restriction 2. For $j \in \{1, \ldots, \ell\}$, let $S^j_0 = \{s \in S \mid \forall \sigma \in \Sigma^{\mathcal{M}}\colon \mathrm{E}^{\mathcal{M}}_\sigma(\mathbf{R}_j \Diamond G_j) = 0\}$ and $S^j_? = \{s \in S \setminus S^j_0 \mid s$ can be reached from $s_I$ without visiting a state in $S^j_0\}$. These sets can be obtained a priori by analyzing the graph structure of $\mathcal{M}$ [4]. Moreover, we consider upper bounds $U^j_s \in \mathbb{Q}$ for the expected reward at state $s \in S^j_?$ such that $U^j_s \geq \max_{\sigma \in \Sigma^{\mathcal{M}}} \mathrm{E}^{\mathcal{M}_s}_\sigma(\mathbf{R}_j \Diamond G_j)$. We compute such upper bounds using single-objective model checking techniques [4,5]. The MILP encoding applies the characterization of expected rewards for MCs as a *linear equation system* [4].

**Lemma 3.** *For every $\sigma \in \Sigma^{\mathcal{M}}_{\mathrm{PS}}$, the following equation system has a unique solution $\Phi\colon \{x_s \mid s \in S\} \to \mathbb{R}^{|S|}$ satisfying $\Phi(x_s) = \mathrm{E}^{\mathcal{M}_s}_\sigma(\mathbf{R}_j \Diamond G_j)$:*

$$\forall s \in S^j_0\colon\ x_s = 0 \qquad \forall s \in S^j_?\colon\ x_s = \sum_{s' \in S} \mathbf{P}(s, \sigma(s), s') \cdot \big(x_{s'} + \mathbf{R}(s, \sigma(s), s')\big)$$

$\forall\, s \in S$: $\triangleright$ *Select an action at each state*

$$\forall\, \alpha \in Act(s): \quad a_{s,\alpha} \in \{0,1\} \tag{1}$$

$$\sum_{\alpha \in Act(s)} a_{s,\alpha} = 1 \tag{2}$$

$\forall\, j \in \{1,\dots,\ell\}$ : $\triangleright$ *Compute expected reward values*

$$\forall\, s \in S_0^j: \quad x_s^j = 0 \tag{3}$$

If $\psi_j$ is maximising, $\pm = +$ and $[\min] = 0$. Otherwise, $\pm = -$ and $[\min] = 1$.

$$\forall\, s \in S_?^j: \quad \pm\, x_s^j \in [0, U_s^j] \tag{4}$$

$$\forall\, \alpha \in Act(s): \quad \pm\, x_{s,\alpha}^j \in [0, U_s^j] \tag{5}$$

$$x_{s,\alpha}^j \leq \sum_{s' \in S} \mathbf{P}(s,\alpha,s') \cdot \left( x_{s'}^j \pm \mathbf{R}_j(s,\alpha,s') \right) \tag{6}$$

$$x_{s,\alpha}^j \leq U_s^j \cdot (a_{s,\alpha} - [\min]) \tag{7}$$

$$x_s^j \leq \sum_{\alpha \in Act(s)} x_{s,\alpha}^j + [\min] \cdot (|Act(s)| - 1) \cdot U_s^j \tag{8}$$

$$\pm\, x_{s_I}^j \sim_j \boldsymbol{p}[\![j]\!] \quad \triangleright \textit{Assert value at initial state} \tag{9}$$

Figure 2: `MILP` encoding for unichain MDP and finite rewards.

*Proof.* Since $\mathcal{M}$ is unichain and we do not collect infinite reward, the only EC of $\mathcal{M}$ (i.e., the only BSCC of $\mathcal{M}^\sigma$ for any $\sigma$) either contains a goal state or only contains transitions with zero reward. It follows that $\forall\, \sigma \in \Sigma_{\mathrm{PS}}^{\mathcal{M}}\colon\ \Pr_\sigma^{\mathcal{M}}(\lozenge S_0^j) = 1$. Lemma 3 follows by standard arguments for MCs with rewards [4, Section 10.5.1].

We discuss the intuition of each constraint in Fig. 2. Let $\Phi\colon Var \to \mathbb{R}$ be an assignment of the occurring variables $Var$ to values. $\Phi$ is a solution of the constraints if all (in)equations are satisfied upon replacing all variables $v$ by $\Phi(v)$.

Lines 1 and 2 encode a strategy $\sigma \in \Sigma_{\mathrm{PS}}^{\mathcal{M}}$ by considering a binary variable $a_{s,\alpha}$ for each state $s$ and enabled action $\alpha$ such that $\sigma(s)(\alpha) = 1$ iff $\Phi(a_{s,\alpha}) = 1$ for a solution $\Phi$. Due to Line 2, exactly one action has to be chosen at each state.

Lines 3 to 8 encode for each objective $\psi_j$ the expected rewards obtained for the encoded strategy $\sigma$. For every $s \in S$, the variable $x_s^j$ represents a (lower or upper) bound on the expected reward at $s$. Line 3 sets this value for all $s \in S_0^j$, reflecting the analogous case from Lemma 3. For $s \in S_?^j$, we distinguish maximizing ($\sim_j = \geq$) and minimizing ($\sim_j = \leq$) objectives $\psi_j$.

For maximizing $\psi_j$, we have $\Phi(x_s^j) \leq \mathrm{E}_\sigma^{\mathcal{M}_s}(\mathbf{R}_j \lozenge G_j)$ for every solution $\Phi$. This is achieved by considering a variable $x_{s,\alpha}^j$ for each enabled action $\alpha \in Act(s)$. In Line 6, we use the equation system characterization from Lemma 3 to assert that the value of $x_{s,\alpha}^j$ can not be greater than the expected reward at $s$, given that the encoded strategy $\sigma$ selects $\alpha$. If $\sigma$ does not select $\alpha$ (i.e., $\Phi(a_{s,\alpha}) = 0$), Line 7 implies $\Phi(x_{s,\alpha}^j) = 0$. Otherwise, this constraint has no effect. Line 8 ensures that every solution satisfies $\Phi(x_s^j) \leq \Phi(x_{s,\alpha}^j) \leq \mathrm{E}_\sigma^{\mathcal{M}_s}(\mathbf{R}_j \lozenge G_j)$ for $\alpha$ with $\Phi(a_{s,\alpha}) = 1$.

For minimizing $\psi_j$, we have $-\Phi(x_s^j) \geq \mathrm{E}_\sigma^{\mathcal{M}_s}(\mathbf{R}_j \lozenge G_j)$ for every solution $\Phi$, i.e., we consider the negated reward values. The encoding is as for maximizing objectives. However, Line 7 yields $\Phi(x^j_{s,\alpha}) = -U^j_s$ if $\alpha$ is not selected. Thus, in Line 8 we add $U^j_s$ for each of the $(|Act(s)| - 1)$ non-selected actions.

Line 9 and our observations above yield $\mathrm{E}^{\mathcal{M}_s}_\sigma(\mathbf{R}_j \Diamond G_j) \geq \Phi(x^j_{s_I}) \geq \boldsymbol{p}[\![j]\!]$ for maximizing and $\mathrm{E}^{\mathcal{M}_s}_\sigma(\mathbf{R}_j \Diamond G_j) \leq -\Phi(x^j_{s_I}) \leq \boldsymbol{p}[\![j]\!]$ for minimizing objectives. Therefore, $\boldsymbol{p}$ is achievable if a solution $\Phi$ exists. On the other hand, if $\boldsymbol{p}$ is achievable by some $\sigma \in \Sigma^{\mathcal{M}}_{\mathrm{PS}}$, the solution $\Phi$ exists with $\Phi(a_{s,\alpha}) = \sigma(s)(\alpha)$, $\Phi(x^j_s) = \Phi(x^j_{s,\alpha}) = \pm \mathrm{E}^{\mathcal{M}_s}_\sigma(\mathbf{R}_j \Diamond G_j)$ if $\alpha = \sigma(s)$, and $\Phi(v) = 0$ for other $v \in Var$.

**Theorem 2.** *For unichain $\mathcal{M}$ and finite rewards, the constraints in Fig. 2 are feasible iff $\boldsymbol{p} \in Ach^{\mathcal{M}}_{\mathrm{PS}}(\mathcal{Q})$.*

**Proposition 1.** *The* `MILP` *encoding above considers $\mathcal{O}(|S| \cdot |Act| \cdot \ell)$ variables.*

### 3.4 Alternative Encoding for Total Rewards

We now consider `PSMA` instances where all objectives $\psi_j = \mathbb{E}_{\sim_j}(\mathbf{R}_j \Diamond G_j)$ are expected *total* reward objectives, i.e., $G_j = \emptyset$. For such instances, we can employ an encoding from [23] (restated in Lemma 4) for `GMA`. In fact, we can often translate reachability reward objectives to total reward objectives, e.g., if the set of goal states can not be left or if all objectives consider the same goal states.

**Lemma 4 ([23]).** *For $S_0 \subseteq S$, let $\Phi\colon Var \to \mathbb{R}_{\geq 0}$ be an assignment of variables $Var = \{y_{s,\alpha} \mid s \in S \setminus S_0, \alpha \in Act(s)\}$ and let $\sigma_\Phi$ be a stationary strategy satisfying $\sigma_\Phi(s)(\alpha) = \Phi(y_{s,\alpha}) / \sum_{\beta \in Act(s)} \Phi(y_{s,\beta})$ for all $s \in S \setminus S_0$ and $\alpha \in Act(s)$ for which the denominator is non-zero. Then, $\Phi$ is a solution to the equation system*

$$\forall\, s \in S \setminus S_0\colon \sum_{\alpha \in Act(s)} y_{s,\alpha} = [s = s_I] + \sum_{\langle s',\alpha' \rangle \in pre(s)} \mathbf{P}(s',\alpha',s) \cdot y_{s',\alpha'}$$

$$1 = \sum_{y_{s,\alpha} \in Var} y_{s,\alpha} \cdot \sum_{s' \in S_0} \mathbf{P}(s,\alpha,s')$$

*iff $\mathrm{Pr}^{\mathcal{M}}_{\sigma_\Phi}(\Diamond S_0) = 1$ and $\forall\, y_{s,\alpha} \in Var\colon \Phi(y_{s,\alpha}) = \mathrm{E}^{\mathcal{M}}_{\sigma_\Phi}(\mathbf{R}_{s,\alpha} \Diamond S_0)$ with reward structure $\mathbf{R}_{s,\alpha}$ given by $\mathbf{R}_{s,\alpha}(\hat{s},\hat{\alpha},s') = [\hat{s} = s \text{ and } \hat{\alpha} = \alpha]$.*

In [23], the lemma is applied to decide achievability of multiple total reward objectives under strategies that are stationary, but not necessarily pure. Intuitively, $\mathrm{E}^{\mathcal{M}}_{\sigma_\Phi}(\mathbf{R}_{s,\alpha} \Diamond S_0)$ coincides with the expected number of times action $\alpha$ is taken at state $s$ until $S_0$ is reached. Since this value can be infinite if $\mathrm{Pr}^{\mathcal{M}}_{\sigma_\Phi}(\Diamond S_0) < 1$, a solution $\Phi$ can only exist if it induces a strategy that almost surely reaches $S_0$.

The encoding for unichain MDP with finite rewards and total reward objectives is shown in Fig. 3, where $S_0 = \bigcap_j S^j_0$ and $S_? = S \setminus S_0$. We consider the constraints in conjunction with Lines 1 and 2 from Fig. 2. Let $\Phi$ be a solution and let $\sigma$ be the strategy encoded by such a solution, i.e., $\sigma(s)(\alpha) = \Phi(a_{s,\alpha})$.

Lines 10 to 12 reflect the equations of Lemma 4. Since $\mathcal{M}$ is unichain and we assume finite rewards, there is just one end component in which no reward can be collected. Hence, $S_0$ is almost surely reached. Line 10 ensures that the

$$\forall s \in S_?, \alpha \in Act(s): \quad y_{s,\alpha} \in [0, V_s \cdot a_{s,\alpha}] \tag{10}$$

$$\sum_{\alpha \in Act(s)} y_{s,\alpha} = [s = s_I] + \sum_{\langle s',\alpha' \rangle \in pre(s)} \mathbf{P}(s', \alpha', s) \cdot y_{s',\alpha'} \tag{11}$$

$$1 = \sum_{s \in S_?} \sum_{\alpha \in Act(s)} y_{s,\alpha} \cdot \sum_{s' \in S_0} \mathbf{P}(s, \alpha, s') \tag{12}$$

$$\forall j \in \{1, \dots, \ell\}: \quad x^j_{s_I} = \sum_{s \in S_?} \sum_{\alpha \in Act(s)} y_{s,\alpha} \cdot \sum_{s' \in S} \big(\mathbf{P}(s, \alpha, s') \cdot \mathbf{R}_j(s, \alpha, s')\big) \tag{13}$$

$$x^j_{s_I} \sim_j \boldsymbol{p}[\![j]\!] \tag{14}$$

Figure 3: MILP encoding for total reward objectives.

strategy in Lemma 4 coincides with the encoded pure strategy $\sigma$. We write $V_s$ for an upper bound of the value a solution can possibly assign to $y_{s,\alpha}$, i.e., $\forall \sigma \in \Sigma^{\mathcal{M}}_{\mathrm{PS}}\colon V_s \geq \mathrm{E}^{\mathcal{M}}_{\sigma}(\mathbf{R}_{s,\alpha} \Diamond S_0)$. Such an upper bound can be computed based on ideas of [5]. More details are given in [17, App. A].

With Lemma 4 we get that $\Phi(y_{s,\sigma(s)})$ is the expected number of times state $s$ is visited under strategy $\sigma$. Therefore, in Line 13 we sum up for each state $s \in S_?$ the expected amount of reward collected at $s$. This yields $\Phi(x^j_{s_I}) = \mathrm{E}^{\mathcal{M}}_{\sigma}(\mathbf{R}_j \Diamond G_j)$. Finally, Line 14 asserts that the resulting values exceed the thresholds given by $\boldsymbol{p}$.

**Theorem 3.** *For unichain $\mathcal{M}$, finite rewards, and total reward objectives, the constraints in Fig. 3 and Lines 1 and 2 of Fig. 2 are feasible iff $\boldsymbol{p} \in Ach^{\mathcal{M}}_{\mathrm{PS}}(\mathcal{Q})$.*

**Proposition 2.** *The* MILP *encoding above considers $\mathcal{O}(|S| \cdot |Act| + \ell)$ variables.*

The encoding for total reward objectives considers fewer variables compared to the encoding of Sect. 3.3 (cf. Proposition 1). In practice, this often leads to faster solving times as we will see in Sect. 6.

### 3.5 Extension to Multichain MDP

We now lift the restriction to unichain MDP, i.e., we consider multichain MDP with finite rewards. We focus on the encoding of Sect. 3.3. Details for the approach of Sect. 3.4 are in [17, App. C]. The key challenge is that the equation system in Lemma 3 does not yield a unique solution for multichain MDP.

*Example 2.* For the multichain MDP in Fig. 5a with $G = \{s_1\}$ we have $S_0 = \{s_1\}$ and $S_? = \{s_0\}$ (the superscript $j$ is omitted as there is only one objective). For $\sigma$ with $\sigma(s_0) = \alpha$ we get $\mathrm{E}^{\mathcal{M}}_{\sigma}(\mathbf{R} \Diamond G) = 0$, but every $\Phi\colon \{x_{s_0}, x_{s_1}\} \to \mathbb{R} \times \{0\}$ is a solution for the equation system in Lemma 3.

For multichain MDP it can be the case that for some strategy $\sigma$ the set $S^j_0$ is not reached with probability 1, i.e., there is a positive probability to stay in the set $S^j_?$ forever. For the induced Markov chain $\mathcal{M}^\sigma$, this means that there is a reachable BSCC consisting only of states in $S^j_?$. Since BSCCs of $\mathcal{M}^\sigma$ coincide with end components of $\mathcal{M}$, we need to inspect the ECs of $\mathcal{M}$ that only consist of

$$
\begin{array}{llr}
\forall\, j \in \{1,\dots,\ell\}, \mathcal{E} \in \mathit{MECS}(\mathcal{M}[\![\mathcal{E}^j_?]\!]): & & \triangleright\ \textit{Detect states with zero reward} \\
\quad \forall\, s \in S[\![\mathcal{E}]\!]: & \pm x^j_s \le U^j_s \cdot (1 - e^j_s) & (15) \\
\quad \forall\, \langle s,\alpha\rangle \in \mathcal{E}: & e^j_{s,\alpha} \in \{0, a_{s,\alpha}\} & (16) \\
\quad\quad \forall\, s' \in \mathit{succ}(s,\alpha): & e^j_{s,\alpha} \le e^j_{s'} & (17) \\
\quad \forall\, s \in S[\![\mathcal{E}]\!]: & e^j_s = \sum_{\alpha \in \mathit{Act}(s)} [\langle s,\alpha\rangle \in \mathcal{E}] \cdot e^j_{s,\alpha} & (18) \\
\quad\quad \forall\, \alpha \in \mathit{Act}(s): & z^j_{s,\alpha} \in [0, V_s \cdot a_{s,\alpha}] & (19) \\
 & z^j_{s,\perp} \in [0, V_s \cdot e^j_s] & (20) \\
\multicolumn{2}{r}{z^j_{s,\perp} + \sum_{\alpha \in \mathit{Act}(s)} z^j_{s,\alpha} = \frac{1}{|S[\![\mathcal{E}]\!]|} + \sum_{\langle s',\alpha'\rangle \in \mathit{pre}(s) \cap \mathcal{E}} \mathbf{P}(s',\alpha',s) \cdot z^j_{s',\alpha'}} & (21) \\
\multicolumn{2}{r}{1 = \sum_{s \in S[\![\mathcal{E}]\!]} \Big( z^{\psi_j}_{s,\perp} + \sum_{\alpha \in \mathit{Act}(s)} [\langle s,\alpha\rangle \notin \mathcal{E}] \cdot z^j_{s,\alpha} \Big)} & (22)
\end{array}
$$

Figure 4: `MILP` encoding for detection of end components.

$S^j_?$-states. These ECs correspond to the ECs of the sub-MDP $\mathcal{M}[\![\mathcal{E}^j_?]\!]$, where $\mathcal{E}^j_?$ is the largest subset of $S^j_? \times \mathit{Act}$ that is closed for $\mathcal{M}$. For each $\mathcal{E} \in \mathit{MECS}(\mathcal{M}[\![\mathcal{E}^j_?]\!])$, we need to detect whether the encoded strategy induces a BSCC $\mathcal{E}' \subseteq \mathcal{E}$.

To cope with multiple ECs, we consider the constraints from Fig. 2 in conjunction with the constraints from Fig. 4. Let $\varPhi$ be a solution to these constraints and let $\sigma$ be the encoded strategy $\sigma$ with $\sigma(s)(\alpha) = \varPhi(a_{s,\alpha})$. For each objective $\psi_j$ and state $s$, a binary variable $e^j_s$ is set to 1 if $s$ lies on a BSCC of the induced MC $\mathcal{M}^\sigma$. We only need to consider states $s \in S[\![\mathcal{E}]\!]$ for $\mathcal{E} \in \mathit{MECS}(\mathcal{M}[\![\mathcal{E}^j_?]\!])$.

Line 15 ensures that the value of $x^j_s$ is set to 0 if $s$ lies on a BSCC of $\mathcal{M}^\sigma$. Lines 16 to 18 introduce binary variables $e^j_{s,\alpha}$ for each state-action pair in the EC such that any solution $\varPhi$ satisfies $\varPhi(e^j_{s,\alpha}) = 1$ iff $\varPhi(e^j_s) = \varPhi(a_{s,\alpha}) = 1$. Line 17 yields that $\varPhi(e^j_{s,\alpha}) = 1$ implies $\varPhi(e^j_{s'}) = 1$ for all successors $s'$ of $s$ and the selected action $\alpha$. Hence, for all $s$ with $\varPhi(e^j_s) = 1$ and for all $s'$ reachable from $s$ in $\mathcal{M}^\sigma$, we have $\varPhi(e^j_{s'}) = 1$ and $\langle s', \sigma(s')\rangle \in \mathcal{E}$. Therefore, we can only set $e^j_s$ to 1 if there is a BSCC $\mathcal{E}' \subseteq \mathcal{E}$ that either contains $s$ or that is almost surely reached from $s$ without leaving $\mathcal{E}$. As finite rewards are assumed, $\mathcal{E}$ can not contain a transition with positive reward, yielding $\mathrm{E}^{\mathcal{M}}_\sigma(\mathbf{R}_j \Diamond G_j) = 0$ if $\varPhi(e^j_s) = 1$.

An assignment that sets all variables $e^j_s$ and $e^j_{s,\alpha}$ to 0 trivially satisfies the constraints in Lines 15 to 18. In Lines 19 to 22 we therefore ensure that if a BSCC $\mathcal{E}' \subseteq \mathcal{E}$ exists in $\mathcal{M}^\sigma$, $\varPhi(e^j_s) = 1$ holds for at least one $s \in S[\![\mathcal{E}']\!]$. The idea is based on the observation that if a BSCC $\mathcal{E}' \subseteq \mathcal{E}$ exists, there is a state $s \in S[\![\mathcal{E}]\!]$ that does not reach the set $S \setminus S[\![\mathcal{E}]\!]$ almost surely. We consider the MDP $\mathcal{M}^{\mathcal{E}}$, a mild extension of $\mathcal{M}[\![\mathcal{E}]\!]$ given by $\mathcal{M}^{\mathcal{E}} = (S[\![\mathcal{E}]\!] \uplus \{s^{\mathcal{E}}_I, s^{\mathcal{E}}_\perp\}, \mathit{Act} \uplus \{\alpha_I, \perp\}, \mathbf{P}^{\mathcal{E}}, s^{\mathcal{E}}_I)$, where $\mathbf{P}^{\mathcal{E}}$ extends $\mathbf{P}[\![\mathcal{E}]\!]$ such that $\mathbf{P}^{\mathcal{E}}(s^{\mathcal{E}}_\perp, \perp, s^{\mathcal{E}}_\perp) = 1$ and $\forall\, s \in S[\![\mathcal{E}]\!]$:

- $\mathbf{P}^{\mathcal{E}}(s^{\mathcal{E}}_I, \alpha_I, s) = 1/|S[\![\mathcal{E}]\!]|$, $\mathbf{P}^{\mathcal{E}}(s, \perp, s^{\mathcal{E}}_\perp) = 1$, and
- $\forall\, \alpha \in \{\hat{\alpha} \in \mathit{Act}(s) \mid \langle s, \hat{\alpha}\rangle \notin \mathcal{E}\} : \mathbf{P}^{\mathcal{E}}(s, \alpha, s^{\mathcal{E}}_\perp) = 1$.

Lines 21 and 22 reflect the equation system from Lemma 4 for MDP $\mathcal{M}^{\mathcal{E}}$ and $S_0 = \{s_\perp\}$. Additionally, Lines 19 and 20 exclude negative solutions and assert

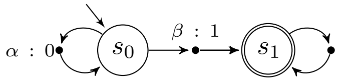

(a) Multichain MDP

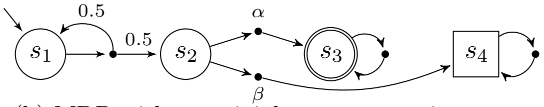

(b) MDP with nontrivial memory requirements

Figure 5: MDPs referenced in Examples 2 and 4.

$\Phi(z^j_{s,\alpha}) = 0$ if $\Phi(a_{s,\alpha}) = 0$ and $\Phi(z^{\psi_j}_{s,\perp}) = 0$ if $\Phi(e^j_s) = 0$ for any solution $\Phi$. Hence, for states $s \in S[\![\mathcal{E}]\!]$ where $\Phi(e^j_s) = 0$, the strategy $\sigma$ encoded by the variables $a_{s,\alpha}$ coincides with the strategy considered in Lemma 4. Assume that solution $\Phi$ yields a BSCC within the states of $\mathcal{E}$ in $\mathcal{M}^\sigma$ and therefore also a BSCC in $(\mathcal{M}^{\mathcal{E}})^\sigma$. Since $s^{\mathcal{E}}_\perp$ has to be reached almost surely in $\mathcal{M}^{\mathcal{E}}$ (cf. Lemma 4), the BSCC has to contain at least one state $s$ with $\Phi(e^j_s) = 1$.

In summary, Lines 19 to 22 imply that every BSCC $\mathcal{E}' \subseteq \mathcal{E}$ of $\mathcal{M}^\sigma$ contains at least one state $s$ with $\Phi(e^j_s) = 1$. Then, with Lines 16 to 18 we get that $\Phi(e^j_{s'}) = 1$ has to hold for *all* $s' \in S[\![\mathcal{E}']\!]$. In $\mathcal{M}^\sigma$, the set $S^j_0 \cup \{s \mid \Phi(e^j_s) = 1\}$ is therefore reached almost surely and all the states in this set get assigned value 0. In this case, the solution of the equation system from Lemma 3 becomes unique again.

**Theorem 4.** *For finite rewards, the constraints in Figs. 2 and 4 are feasible iff* $\boldsymbol{p} \in Ach^{\mathcal{M}}_{\mathrm{PS}}(\mathcal{Q})$.

### 3.6 Extension to Infinite Rewards

Our approach can be modified to allow `PSMA` instances where infinite expected reward can be collected, i.e., where Restriction 2 does not hold. Infinite reward can be collected if we cycle through an EC of $\mathcal{M}$ that contains a transition with positive reward. Such instances are of practical interest as this often corresponds to strategies that do not accomplish a certain goal (e.g., a robot that stands still and therefore requires infinite time to finish its task).

We sketch the necessary modifications. More details are in [17, App. B]. Let $S_\infty$ be the set of states where every pure strategy induces infinite reward for at least one minimizing objective. To ensure that the `MILP` instance has a (real-valued) solution, we consider the sub-MDP of $\mathcal{M}$ obtained by removing $S_\infty$.

If infinite reward can be collected in an EC, it should not be considered in Fig. 4. We therefore let $\mathcal{E}$ range over maximal ECs that only consist of (a) states in $S^j_?$ and (b) transitions with reward 0.

The upper bounds $U^j_s$ for the maximal expected rewards at each state can not be set to $\infty$. However, for the encoding it suffices to compute values that are sufficiently large. However, we remark that in practice our approach from [17, App. B] can lead to very large values, yielding numerical instabilities.

For maximizing objectives, we introduce one additional objective which, in a nutshell, checks that the probability to reach a 0-reward BSCC is below 1. If this is the case, there is a positive probability to reach a BSCC in which infinitely many reward can be collected.

## 4 Computing the Pareto Front

Our next goal is to compute the *pure stationary Pareto front* $Pareto_{\mathrm{PS}}^{\mathcal{M}}(\mathcal{Q})$ for MDP $\mathcal{M}$ and multi-objective query $\mathcal{Q}$. This set can be very large, in particular if the objectives are strongly conflicting with many different tradeoffs. In the worst case, every pure stationary strategy induces a point $\boldsymbol{p} \in Pareto_{\mathrm{PS}}^{\mathcal{M}}(\mathcal{Q})$ (e.g., for $\mathcal{Q} = \langle \mathbb{E}_{\leq}(\mathbf{R}\Diamond G), \mathbb{E}_{\geq}(\mathbf{R}\Diamond G)\rangle$). We try to find an approximation of $Pareto_{\mathrm{PS}}^{\mathcal{M}}(\mathcal{Q})$.

**Definition 7.** *Let* $\boldsymbol{\epsilon} \in (\mathbb{R}_{>0})^\ell$. *An* $\boldsymbol{\epsilon}$-approximation *of* $P \subseteq (\mathbb{R}_\infty)^\ell$ *is a pair* $\langle L, U\rangle$ *with* $L \subseteq P \subseteq U$ *and* $\forall \boldsymbol{p} \in P\colon \exists \boldsymbol{p}' \in L \cup ((\mathbb{R}_\infty)^\ell \setminus U)\colon |\boldsymbol{p} - \boldsymbol{p}'| \leq \boldsymbol{\epsilon}$.

| Pure Stationary Pareto Approximation Problem ($\texttt{PSP}^{\approx}$) | |
|---|---|
| **Input:** | MDP $\mathcal{M}$, $\ell$-dimensional multi-objective query $\mathcal{Q}$, precision $\boldsymbol{\epsilon} \in (\mathbb{R}_{>0})^\ell$ such that $Pareto_{\mathrm{PS}}^{\mathcal{M}}(\mathcal{Q}) \subseteq \mathbb{R}^\ell$ |
| **Output:** | An $\boldsymbol{\epsilon}$-approximation of $cl^{\mathcal{Q}}(Pareto_{\mathrm{PS}}^{\mathcal{M}}(\mathcal{Q}))$ |

For simplicity, we only consider inputs that satisfy restriction Restriction 2, i.e., for $\psi_j = \mathbb{E}_{\sim_j}(\mathbf{R}_j\Diamond G_j)$ there is $U^j \neq \infty$ such that $\forall\, \sigma \in \Sigma_{\mathrm{PS}}^{\mathcal{M}}\colon\ U^j \geq \mathrm{E}_{\sigma}^{\mathcal{M}}(\mathbf{R}_j\Diamond G_j)$. Ideas of Sect. 3.6 can be used for some other inputs. An all-embracing treatment of infinite rewards, in particular for maximizing $\psi_j$, is subject to future work.

Our approach for $\texttt{PSP}^{\approx}$ successively divides the solution space into candidate regions. For each region $\mathcal{R}$ (initially, let $\mathcal{R} = [0, U^1] \times \cdots \times [0, U^\ell]$), we use the $\texttt{MILP}$ encoding from Sect. 3 with an optimization function to find a point $\boldsymbol{p} \in \mathcal{R} \cap Pareto_{\mathrm{PS}}^{\mathcal{M}}(\mathcal{Q})$ (or find out that no such point exists). The region $\mathcal{R}$ is divided into (i) an achievable region $\mathcal{R}_{\mathrm{A}} \subseteq Ach_{\mathrm{PS}}^{\mathcal{M}}(\mathcal{Q})$, (ii) an unachievable region $\mathcal{R}_{\mathrm{U}} \subseteq \mathbb{R}^\ell \setminus Ach_{\mathrm{PS}}^{\mathcal{M}}(\mathcal{Q})$, (iii) further candidate regions $\mathcal{R}_1, \dots, \mathcal{R}_n$ that are analyzed subsequently, and (iv) the remaining area $\mathcal{R} \setminus (\mathcal{R}_{\mathrm{A}} \cup \mathcal{R}_{\mathrm{U}} \cup \mathcal{R}_1 \cup \cdots \cup \mathcal{R}_n)$ which does not require further analysis as we are only interested in an $\boldsymbol{\epsilon}$-approximation. The procedure stops as soon as no more candidate regions are found.

*Example 3.* Fig. 6 sketches the approach for an MDP $\mathcal{M}$ and a query $\mathcal{Q}$ with two maximizing objectives. We maintain a set of achievable points (light green) and a set of unachievable points (red). Initially, our candidate region corresponds to $\mathcal{R}_1 = [0, U^1] \times [0, U^2]$ given by the white area in Fig. 6a. We consider the direction vector $\boldsymbol{w}_1$ which is orthogonal to the line connecting $\langle U^1, 0\rangle$ and $\langle 0, U^2\rangle$. To find some point $\boldsymbol{p} \in Pareto_{\mathrm{PS}}^{\mathcal{M}}(\mathcal{Q}) \cap \mathcal{R}_1$, we solve the $\texttt{MILP}$ resulting from the constraints as in Sect. 3, the constraint $\langle x_{s_I}^1, x_{s_I}^2\rangle \in \mathcal{R}_1$, and the optimization function $\boldsymbol{w}_1 \cdot \langle x_{s_I}^1, x_{s_I}^2\rangle$. Fig. 6b shows the obtained point $\boldsymbol{p}_1 \in \mathcal{R}_1$. Since $\boldsymbol{p}_1$ is achievable, we know that any point in $cl^{\mathcal{Q}}(\{\boldsymbol{p}_1\})$ has to be achievable as well. Moreover, the set $\{\boldsymbol{p} \in \mathcal{R}_1 \mid \boldsymbol{w}_1 \cdot \boldsymbol{p} > \boldsymbol{w}_1 \cdot \boldsymbol{p}_1\}$ indicated by the area above the diagonal line in Fig. 6b can not contain an achievable point. The gray areas do not have to be checked in order to obtain an $\boldsymbol{\epsilon}$-approximation. We continue with $\mathcal{R}_2$ indicated by the white area and the direction vector $\boldsymbol{w}_2$, orthogonal to the line connecting $\langle 0, U^2\rangle$ and $\boldsymbol{p}_1$. As before, we solve an $\texttt{MILP}$ now yielding the point $\boldsymbol{p}_2$ in Fig. 6c. We find achievable points $cl^{\mathcal{Q}}(\{\boldsymbol{p}_2\})$ but no further unachievable points. The next iteration considers candidate region $\mathcal{R}_3$ and direction vector

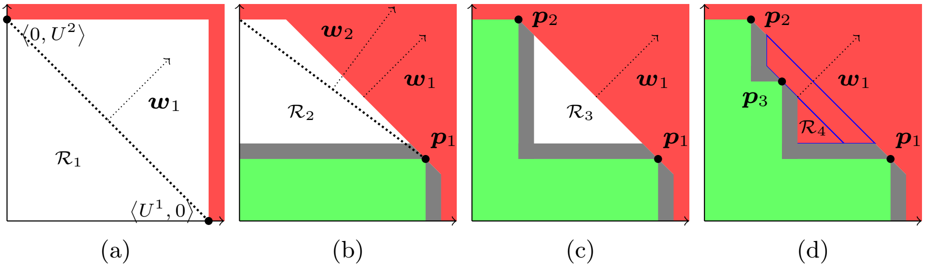

Figure 6: Example exploration of achievable points.

$\boldsymbol{w}_1$, yielding point $\boldsymbol{p}_3$ shown in Fig. 6d. The trapezoidal area is added to the unachievable points whereas $cl^{\mathcal{Q}}(\{\boldsymbol{p}_3\})$ is achievable. Finally, we check $\mathcal{R}_4$ for which the corresponding MILP instance is infeasible, i.e., $\mathcal{R}_4$ is unachievable.

The ideas sketched above can be lifted to $\ell > 2$ objectives. Inspired by [24, Alg. 4], we choose direction vectors that are orthogonal to the convex hull of the achievable points found so far. In fact, for total reward objectives we can apply the approach of [24] to compute the points in $Pareto^{\mathcal{M}}_{\mathrm{PS}}(\mathcal{Q}) \cap Pareto^{\mathcal{M}}(\mathcal{Q})$ first and only perform MILP-solving for the remaining regions. As the distance between two found points $\boldsymbol{p}, \boldsymbol{p}'$ is at least $|\boldsymbol{p} - \boldsymbol{p}'| \geq \boldsymbol{\epsilon}$, we can show that our approach terminates after finding at most $\prod_j U^j / \boldsymbol{\epsilon}[\![j]\!]$ points. Other strategies for choosing direction vectors are possible and can strongly impact performance.

## 5 Bounded Memory

For GMA, it is necessary and sufficient to consider strategies that require memory exponential in the number of objectives [20,24,40] by storing which goal state set has been reached already. In contrast, restricting to pure (but not necessarily stationary) strategies imposes nontrivial memory requirements that do not only depend on the number of objectives, but also on the point that is to be achieved.

*Example 4.* Let $\mathcal{M}$ be the MDP in Fig. 5b and $\mathcal{Q} = \langle \mathbb{P}_{\geq} (\Diamond G_{\circledcirc}), \mathbb{P}_{\geq} (\Diamond G_{\square}) \rangle$. The point $\boldsymbol{p}_k = \langle 0.5^k, 1{-}0.5^k \rangle$ for $k \in \mathbb{N}$ is achievable by taking $\alpha$ with probability $0.5^k$. $\boldsymbol{p}_k$ is also achievable with the *pure* strategy $\sigma_k$ where $\sigma_k(\hat{\pi}) = \alpha$ iff $|\hat{\pi}| \geq k$. $\sigma_k$ uses $k$ memory states. Pure strategies with fewer memory states do not suffice.

We search for pure strategies with *bounded memory*. For an MDP $\mathcal{M}$ and $K > 0$, let $\Sigma^{\mathcal{M}}_{\mathrm{P},K}$ denote the set of pure $K$-memory strategies, i.e., any $\sigma \in \Sigma^{\mathcal{M}}_{\mathrm{P},K}$ can be represented by a *Mealy machine* using up to $K$ states (c.f. [17, App. D]). For a query $\mathcal{Q}$, let $Ach^{\mathcal{M}}_{\mathrm{P},K}(\mathcal{Q})$ be the set of points achievable by some $\sigma \in \Sigma^{\mathcal{M}}_{\mathrm{P},K}$.

| Pure Bounded Multi-objective Achievability Problem (PBMA) |
|---|
| **Input:** MDP $\mathcal{M}$, multi-objective query $\mathcal{Q}$, memory bound $K$, point $\boldsymbol{p} \in (\mathbb{R}_{\infty})^{\ell}$ |
| **Output:** Yes iff $\boldsymbol{p} \in Ach^{\mathcal{M}}_{\mathrm{P},K}(\mathcal{Q})$ |

Table 1: Results for stationary strategies.

| Bench- | | Instance 1 | | | | $\varepsilon$=0.01 | | $\varepsilon$=0.001 | | Instance 2 | | | | $\varepsilon$=0.01 | | $\varepsilon$=0.001 | |
|---|---|---|---|---|---|---|---|---|---|---|---|---|---|---|---|---|---|
| mark | $\ell$ | Par. | $\lvert S\rvert$ | $\%\mathcal{E}$ | $\overline{Act}$ | Time | $\lvert P\rvert$ | Time | $\lvert P\rvert$ | Par. | $\lvert S\rvert$ | $\%\mathcal{E}$ | $\overline{Act}$ | Time | $\lvert P\rvert$ | Time | $\lvert P\rvert$ |
| dpm | 2* | 2 | 1272 | 32 | 3.2 | 17 | 37 | 315 | 377 | 3 | 1696 | 30 | 3.2 | 82 | 30 | TO | |
| eajs | 2* | 2-3 | 689 | 0 | 1.2 | 5 | 23 | 45 | 202 | 3-6 | $2{\cdot}10^4$ | 0 | 1.2 | 201 | 52 | 3787 | 375 |
| jobs | 3* | 3-2 | 17 | 0 | 1.1 | 3 | 3 | 2 | 3 | 5-2 | 117 | 0 | 1.5 | 2042 | 76 | TO | |
| mutex | 3* | 1 | 1795 | 36 | 2.2 | TO | | TO | | 2 | $1{\cdot}10^4$ | 33 | 2.3 | TO | | TO | |
| polling | 2 | 2-2 | 233 | 86 | 1.5 | 6 | 5 | 23 | 6 | 3-2 | 990 | 84 | 1.8 | 299 | 5 | TO | |
| rg | 2* | 2-1-20 | 2173 | 14 | 2.9 | 5 | 5 | 12 | 5 | 5-2-50 | $3{\cdot}10^4$ | 5 | 3.1 | 496 | 27 | TO | |
| rover | 2* | 2500 | $2{\cdot}10^4$ | 0 | 1.2 | 110 | 47 | 417 | 251 | 5000 | $4{\cdot}10^4$ | 0 | 1.2 | 258 | 47 | 3105 | 472 |
| serv | 2* | | $5{\cdot}10^4$ | 93 | 1.9 | 1828 | 38 | TO | | | | | | | | | |
| str | 2* | 30 | 1426 | 0 | 1.3 | 11 | 21 | 822 | 218 | 500 | $4{\cdot}10^5$ | 0 | 1.3 | 2428 | 17 | TO | |
| team2 | 2* | 2 | 1847 | 24 | 1.2 | 2 | 5 | 2 | 5 | 3 | $1{\cdot}10^4$ | 21 | 1.2 | 18 | 43 | MO | |
| team3 | 3* | 2 | 1847 | 24 | 1.2 | 165 | 15 | 166 | 15 | 3 | $1{\cdot}10^4$ | 21 | 1.2 | TO | | TO | |
| uav | 2* | 750 | $2{\cdot}10^5$ | 29 | 1.6 | 400 | 39 | 5799 | 332 | 1000 | $4{\cdot}10^5$ | 31 | 1.8 | 3546 | 36 | TO | |
| wlan | 2* | 0 | 2954 | 0 | 1.3 | 160 | 16 | TO | | 2 | $3{\cdot}10^4$ | 0 | 1.3 | 6728 | 23 | TO | |

The pure bounded Pareto approximation problem is defined similarly. We reduce a PBMA instance to an instance for PSMA. The idea is to incorporate a memory structure of size $K$ into $\mathcal{M}$ and then construct a pure stationary strategy in this product MDP (see, e.g., [29] for a similar construction). The set of strategies can be further refined by considering e.g., a memory structure that only allows counting or that only remembers visits of goal states. See [17, App. D] for details.

## 6 Evaluation

We implemented our approach for $\text{PSP}^{\approx}$ in the model checker Storm [16] using Gurobi [27] as back end for MILP-solving. The implementation takes an MDP (e.g., in Prism syntax), a multi-objective query, and a precision $\varepsilon > 0$ as input and computes an $\boldsymbol{\epsilon}$-approximation of the Pareto front. Here, we set $\boldsymbol{\epsilon}[\![j]\!] = \varepsilon \cdot \delta_j$, where $\delta_j$ is the difference between the maximal and minimal achievable value for objective $\psi_j$. We also support reward objectives for Markov automata via [38]. The computations within Gurobi might suffer from numerical instabilities. To diminish their impact, we use the *exact* engine of Storm to confirm for each MILP solution that the encoded strategy achieves the encoded point. However, sub-optimal solutions returned by Gurobi may still yield inaccurate results.

We evaluate our approach on 13 multi-objective benchmarks from [24,28,38], each considering one or two parameter instantiations. Application areas range over scheduling (dpm [37], eajs [1], jobs [10], polling [43]), planning (rg [6], rover [28], serv [32], uav [21]), and protocols (mutex [38], str [38], team [15], wlan [31]).

The results for pure stationary strategies are summarized in Table 1. For each benchmark we denote the number of objectives $\ell$ and whether the alternative encoding from Sect. 3.4 has been applied (*). For each parameter instantiation (Par.), the number of states ($|S|$), the percentage of the states that are contained in an end component ($\%\mathcal{E}$), and the average number of available actions at each state ($\overline{Act}$) are given. For each precision $\varepsilon \in \{0.01, 0.001\}$, we then depict the

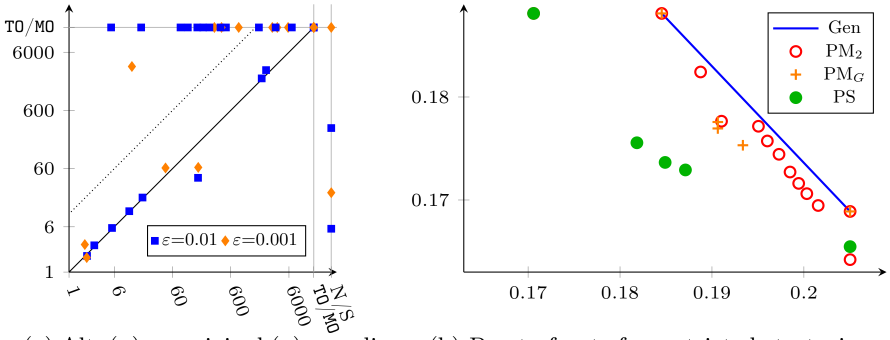

(a) Alt. ($x$) vs. original ($y$) encoding. (b) Pareto fronts for restricted strategies.

Figure 7: Comparison of the two encodings (left) and impact of memory (right).

runtime of Storm and the number of points on the computed approximation of the Pareto front. TO denotes that the approach did not terminate within 2 hours, MO denotes insufficient memory (16 GB). All experiments used 8 cores of an Intel® Xeon® Platinum 8160 Processor.

Storm is often able to compute pure stationary Pareto fronts, even for models with over 100 000 states (e.g., uav). However, the model structure strongly affects the performance. For example, the second instance of jobs is challenging although it only considers 117 states, a low degree of nondeterminism, and no (non-trivial) end components. Small increments in the model size can increase runtimes significantly (e.g., dpm or uav). If a higher precision is requested, much more points need to be found, which often leads to timeouts. Similarly, for more than 2 objectives the desired accuracy can often not be achieved within the time limit. The approach can be stopped at any time to report on the current approximation, e.g., after 2 hours Storm found 65 points for Instance 1 of mutex.

For almost all benchmarks, the objectives could be transformed to total reward objectives, making the more efficient encoding form Sect. 3.4 applicable. We plot the runtimes of the two encoding in Fig. 7a. The alternative encoding is superior for almost every benchmark. In fact, the original encoding timed out for many models as indicated at the horizontal line at the top of the figure.

In Fig. 7b we plot the Pareto front for the first polling instance under general strategies (Gen), pure 2-memory strategies that can change the memory state exactly once ($\mathrm{PM}_2$), pure strategies that observe which goal state set $G_j$ has been visited already ($\mathrm{PM}_G$), and pure stationary strategies (PS). Adding simple memory structures already leads to noticeable improvements in the quality of strategies. In particular, $\mathrm{PM}_2$ strategies perform quite well, and even outperform $\mathrm{PM}_G$ strategies (which would be optimal if randomization were allowed).

*Data availability.* The artifact [18] accompanying this paper contains source code, benchmark files, and replication scripts for our experiments.

*Acknowledgments.* The authors thank Sebastian Junges for his valuable contributions during early stages of this work.
